# Defining the Properties of an Array of –NH_2_-Modified Substrates for the Induction of a Mature Osteoblast/Osteocyte Phenotype from a Primary Human Osteoblast Population Using Controlled Nanotopography and Surface Chemistry

**DOI:** 10.1007/s00223-016-0202-y

**Published:** 2016-10-28

**Authors:** Sandra A. Fawcett, Judith M. Curran, Rui Chen, Nicholas P. Rhodes, Mark F. Murphy, Peter Wilson, Lakshminarayan Ranganath, Jane P. Dillon, James A. Gallagher, John A. Hunt

**Affiliations:** 1 Institute of Ageing and Chronic Disease, University of Liverpool, The William Henry Duncan Building, West Derby Street, Liverpool, L7 8TX UK; 2 School of Engineering, University of Liverpool, Harrison Hughes Building, Liverpool, L69 3GH UK; 3 School of Pharmacy and Biomolecular Sciences, Liverpool John Moores University, Byrom Street, Liverpool, L33AF UK

**Keywords:** Biomaterials, Surface coatings, Amine, Silane, Osteoblast, Osteocyte

## Abstract

Accelerating the integration of a joint replacement or the healing of a bone fracture, particularly a complicated non-union fracture, would improve patient welfare and decrease healthcare costs. Currently, an autologous bone graft is the gold standard method for the treatment of complicated non-union fractures, but it is not always possible to harvest such a graft. A proactive highly inductive so-called smart material approach is pertinent in these cases. In this study, the surface chemistry of a previously approved material with desirable bulk material properties was modified to investigate its potential as an economical and effective alternative. The objective was to create stable synthetic chemical coatings that could guide cells along the osteogenic lineage required to generate mineralised tissue that would induce and accelerate bone healing. Primary human osteoblast-like cells were cultured in vitro for 7, 14 and 28 days on amine-terminated (chain length in the range 3–11) silane-modified glass surfaces with controlled nanotopography, to determine how surface chemistry and nanotopography change osteoblast function. The materials were characterised using atomic force microscopy (AFM), scanning electron microscopy (SEM), water contact angle (WCA) and a novel ninhydrin assay. The cells were analysed using qRT-PCR, von Kossa tinctural staining for mineralisation, and visualised using both transmitted white light and electron microscopy. Bone-like nodules, quantified using microscopy, only formed on the short-chain (chain length 3 and 4) amines after 7 days, as did the up-regulation of sclerostin, suggestive of a more mature osteoblast phenotype. In this paper, we report more rapid nodule formation than has previously been observed, without the addition of exogenous factors in the culture medium. This suggests that the coating would improve the integration of implants with bone or be the basis of a smart biomaterial that would accelerate the bone regeneration process.

**Electronic supplementary material:**

The online version of this article (doi:10.1007/s00223-016-0202-y) contains supplementary material, which is available to authorized users.

## Introduction

Ten to 15% of recorded bone fractures do not heal spontaneously within the first 6–8 weeks and are thus classed as non-union bone fractures, which are associated with an extensive fracture site, rendering the normal bone healing processes inadequate [1]. These fractures present a significant challenge for orthopaedic clinicians and represent an area of modern health care where regenerative strategies could have an immediate and significant short-term impact. The best current treatment is autologous bone graft, which is very successful when sufficient healthy bone is available. The major limitation therein is the availability of donor bone and the associated implications of bone harvesting and patient recovery [2, 3]. One potential solution is the storage of bone for future use, a technique that has a good prognosis when treating chronic diseases [4] but the effectiveness of which is limited for treatment of acute trauma. Joint replacement is also a significant area where biomaterials that could provide increased osteoinduction and osteoconduction would be welcomed.

Thus, the development of such synthetic materials that could be directly implanted into the site of a non-union fracture or site of a joint replacement, and so control the response of mesenchymal stem cells (MSCs) and osteoblasts, could have a dramatic clinical impact. There is extensive research identifying selected surface/material properties that can be used to induce the osteogenic differentiation of MSCs [5–7]. Data derived from these studies have demonstrated that MSCs can respond to stimuli at the submicron scale and are sensitive to changes in chemistry, topography and material modulus. In addition, the beneficial effect of introducing an amine (–NH_2_ or –NH_3_) group to the surface of a substrate to improve the osteoconductive properties of a substrate has been previously been reported [8, 9].

Methods for introducing and enriching substrates with amine functionality are varied, and characterisation of the materials has not fully considered the combinatorial effects of surface chemistry and nanotopography on the response of cells cultured on their surface. In addition, the degree of surface amine enrichment on the deposition of the amine group and the associated change in topographical profile, a direct effect of introducing a chemical group to a surface, has not been considered. Thus, it is the combination of surface chemistry and nanotopography which enhances osteoconduction, i.e. the response of osteoblasts to the surface, and which has not been fully investigated. As the process of natural bone healing differs when autologous bone is introduced (osteoblast-containing bone matrix) [10], it is reasonable to assume that the material properties required for osteoinduction may be different from osteoconduction. Nevertheless, these complex biological interactions must be investigated.

We have previously demonstrated that silane modification is a technique that can be utilised to introduce a chemical group to a surface, i.e. –NH_2_, on an array of base substrates/clinically relevant degradable polymers [11]. Changing the chain length of the silane used in the production of –NH_2_-enriched monolayers is effective for controlling the surface chemistry and associated nanotopography, controlling initial integrin binding, focal adhesion formation and subsequent mechanotransduction events. Silane modification has the potential to mimic the unique properties of bone extracellular matrix (ECM) by the addition of chemical and nanotopographical surface features that are comparable to ECM. Optimisation of these parameters would result in surfaces that enhance the activity of osteoblasts and the production of substrate that could replace the need for autologous bone grafting. These novel materials would be chemically stable and require no manipulation with biological agents prior to implantation, therefore potentially providing cost-effective directly implantable osteoconductive materials that can be stocked and stored for future use (an off-the-shelf material). Surface modifications could be introduced to both particulate- or scaffold-type matrices, in addition to solid substrates (such as metallic implants) as required. This highlights the significant impact the versatility of this approach could have against the need for identification of specific material/surface parameters that could be used to control osteoblast responses and possibly that of other tissues.

This research has investigated a range of –NH_2_-presenting silane-modified layers. The chain length of the silanes was used as a variable to control chemical group deposition, via the formation of a cohesive monolayer and nanotopography. Surfaces amine group density was quantified using a ninhydrin assay, and the hydrophobicity of the surfaces quantified by water contact angle analysis. Topographical profiles were analysed using AFM. Primary human osteoblasts were cultured in contact with the test surfaces, adhesion, morphology, phenotype, and the production of calcified nodules/matrix were examined using light and electron microscopy, and real-time polymerase chain reaction (qRT-PCR) was used to identify markers of osteoblast maturation and osteocytes [12, 13]. All cultures were conducted in basal conditions.

## Methods and Materials

### Preparation and Modification of Borosilicate Glass

Twelve-millimetre-diameter glass coverslips (SLS, UK) were cleaned using 0.5 M sodium hydroxide (Sigma, UK) for 30 min (Fisher, UK), three changes of distilled water and 1 M nitric acid (Sigma, UK) for 30 min followed by three further changes of distilled water (all conducted in an ultrasonic water bath) and dried at 50 °C. Untreated controls were set aside at this point. Glass coverslips were submerged in 0.1 M solutions of the following: (3-aminopropyl)triethoxysilane (CL3) (Sigma, UK), 4-(triethoxyslyl)butan-1-amine (CL4) (Fluorochem), 3-(2-aminoethylamino) propyldimethoxymethylsilane (CL6)(Sigma, UK), N-(6-aminohexyl) amnomethyltriethoxysilane (CL7)(Fluorochem, UK) and 11-aminoundecyltriethoxysilane (CL11)(Fluorochem, UK), for 30 min at room temperature, and then washed with isopropyl alcohol and then distilled water for 5 min.

### Atomic Force Microscopy (AFM)

Modified glass samples and unmodified controls were attached to glass microscope slides using double-sided adhesive tape and examined using an AFM microscope. All images were obtained using an Asylum Research atomic force microscope (MFP-3D). The MFP-3D was coupled to an Olympus IX50 inverted optical (IO) microscope to enable precise positioning of the AFM probe above the sample. The system is placed upon a TS-150 active vibration isolation table (HWL Scientific instruments GmbH, Germany), which was located inside an acoustic isolation enclosure (IGAM GmbH, Germany) to help eliminate external noise. All images were obtained in tapping mode using Olympus TR400PSA cantilevers. The cantilever spring constant was determined using the thermal noise method, which is software driven for the MFP-3D. Four areas from 3 samples of each modification were scanned in tapping mode over a 500-nm scan area.

### Water Contact Angle Measurements

Water contact angles were recorded using a Camtel dynamic contact angle machine (Camtel Ltd. UK). Substrates that had been modified on both sides were immersed into water, and the dynamic contact angle measurements recorded. A total of 6 repeats were recorded for each modification, and results presented as mean ± standard deviation (SD) with the data analysed using ANOVA to test for statistical significance.

### Ninhydrin Assay on Films and Glass

The concentration of surface amine groups was measured using a novel ninhydrin assay, allowing both visualisation and quantification. A clean glass coverslip was submerged in a 0.1 M silane solution for 30 min to apply the –NH2 coating as detailed above. A control untreated coverslip was also used. Subsequently, 1 mL ninhydrin solution (0.35 g of ninhydrin (Sigma, UK) dissolved in 100 mL of ethanol) was added to the materials (including the untreated material) and then heated to 90 °C in an oven for 5 min. The solution was removed from the coverslips and diluted 1:3 with ethanol and then absorbance at 600 nm measured against the untreated control blank. The change in colour of the solution from yellow to purple due to the presence of amine was quantified by reference to a standard curve (ranging from 20 to 120 μM). The coverslips were imaged and photographed using transmitted light microscopy after removal of the ninhydrin reagent to visualise the distribution of the amine groups across the surfaces as purple staining. The concentration of –NH_2_ group deposition was calculated from the loss of amine concentration from the coating solution.

### Isolation of Primary Human Osteoblast-Like Cells

Human osteoblast-like cells were cultured from explants of human bone from ethically approved osteoarthritis surgery (total number of donors was 2—both samples are from trabecular bone below the tibial plateau from knee joint. Both donors were male, and ages were 50 and 62 at time of surgery. In both cases, trabecular bone samples were taken from OA joints, but samples were taken from healthy margin during the OA joint replacement surgery). They were washed with a solution of PBS with streptomycin and penicillin (Sigma, UK). They were then cut into 1- to 2-mm-diameter pieces, and 6–8 of the bone pieces placed in a 9-cm-diameter disposable petri dish, cultured with 10 ml DMEM media plus 5% foetal bovine serum (Sigma, UK). Cultures were incubated for 6–8 weeks, until cells formed a confluent monolayer [14, 15].

### Primary Human Osteoblast-Like Cells on Silane-Modified Glass

5 × 10^4^ (in 100 μL media) of the osteoblast-like cells (isolated as mentioned above) were seeded onto glass test surfaces and incubated at 37 °C for 1 h in a 24-well plate. Following this, 2 ml of DMEM media with 5% foetal bovine serum (Sigma, UK) were added to each well and incubated at 37 °C, 5% CO_2_ for 7, 14 and 28 days. One millilitre of medium was removed and replaced with fresh media at 14 days. At the appropriate time points, samples were removed from culture and analysed by von Kossa staining (calcified extracellular matrix), SEM and qRT-PCR. Cell media were saved at each time point for lactate and glucose measurement (see supplementary materials).

### Lactate and Glucose Measurement in Culture Media (Results in Supplementary Materials)

Lactate and glucose depletion were measured in the culture media using a RAPID Systems blood gas analyser (Siemens, Germany). One millilitre of culture media was injected into the analyser, and readings for glucose and lactate were taken. These markers indicate the proliferation of the cells over the 28-day culture.

### Von Kossa Staining of Coverslips with Primary Human Osteoblast-Like Cells

At the appropriate time point, cells were removed from culture and washed with PBS. Cells were fixed using a 2% formaldehyde and 4% sucrose solution for 15 min at 37 °C and then washed with PBS and distilled water. Samples were transferred to 1% silver nitrite solution for 1 h under UV light. Following this, samples were washed and placed into a 2.5% (w/v) sodium thiosulphate solution for 15 min at room temperature, counterstained with Weigerts haematoxylin for 3 min, washed in tap water for 5 min (Sigma, UK), then mounted on a microscope slide using an aqueous mount (Sigma, UK) and imaged using a AxioVision microscope (Zeiss, Germany). This was repeated 3 times for each time point. Representative images of 7-day cultures are used in results section. Additional images taken at 14 and 28 day are included in supplementary materials.

### Calcified Nodule Count

Nodules were counted for each sample by setting the light microscope to a low magnification (2.5 × objective) and observing the whole sample in one field of view. Nodule count was recorded for each sample (*N* = 16).

### Scanning Electron Microscopy (SEM)

Samples were removed from culture, washed with PBS and fixed using 2.5% (w/v) glutaraldehyde for 15 min at room temperature, washed with PBS and submerged in 70% ethanol for 15 min, then 90% ethanol for 15 min followed by two changes of 100% ethanol for 15 min. Samples were dried using a critical point dryer (Prion, UK). Dry samples were fixed to SEM stubs (Agar Scientific, UK) using double-sided carbon sticky tabs (Agar Scientific, UK). The samples were then coated with 20 nm of chromium using a sputter coater (Emtech, UK). The samples were observed under a Leo 1550 FESEM (Zeiss, UK). This was repeated 3 times for each time point. Representative images were used in results section. Where nodule formation was observed, measurements were taken using the annotation function on the LEO SEM software (Zeiss, Germany).

### Preparation of RNA Using TRIzol

Samples were removed from culture, placed into clean 24-well tissue culture plates (SLS Ltd, UK) and washed using sterile Dulbecco’s PBS (Sigma, UK) to remove any non-adherent cells. Five hundred microlitre TRIzol (Sigma, UK) was added to each well, incubated for 5 min, then removed and frozen at -80 °C until required.

For analysis, samples were defrosted at room temperature and 100 μl chloroform (BDH, UK) was added and then vortex mixed. Each tube was spun at 18,000 g for 5 min, and the upper layer removed into a new tube. Three hundred microlitre isopropanol (Sigma, UK) was added to this new tube and centrifuged at 18,000 g for 15 min. The supernatant was discarded, 500 μl of 100% ethanol was added, and the tube centrifuged for 5 min (18,000 g). The supernatant was again discarded, 200 μl of 70% ethanol was added and the tube spun for 2 min at 18,000 g. The pellet was re-suspended in 10 μl DNA/RNA-free ultrapure water (Sigma, UK). Generic DNA contamination was eliminated using commercially available DNAse kits (Invitrogen, UK) used according to the manufacturer’s instructions. First-strand cDNA was synthesised using a proprietary kit (Invitrogen, UK) following the manufacturer’s instructions.

### qRT-PCR

Quantitative real-time polymerase chain reaction was conducted using primers for osteopontin, osteocalcin, osteonectin, collagen I, CBFA-1 and sclerostin (Fig. 7), with all results normalised against the housekeeping gene β-actin using the ∆∆ct equation. Twenty microlitre forward and 20 μl reverse primer were mixed with 160 μl DNA/RNA-free water. The following reagents were added in triplicate to the wells of a RT-PCR 96-well plate (Bio-Rad, UK): 2 μl cDNA template, 7.5 μl SyBR green (Bio-Rad, UK), 4.5 μl DNA/RNA-free water and 1 μl diluted primer (as above). qRT-PCR was performed using an i-Cycler (Bio-Rad, UK), using previously defined optimum temperatures for each primer (data not included). Lack of contamination of samples was confirmed using standard melt curve analysis. For each sample and each gene, a total of 6 repeats were carried out. Statistical analysis was carried out using ANOVA (Table 1).

**Table 1:** Primers for PCR

Target gene	Primer bases	Temp. in °C
β-Actin	GGACCTGACTGACTACCTCGCCATCTCTTGCTCGAAG	53.9
COL1A1	GCCACTCCAGGTCCTCAGCCACAGCACCAGCAACAC	54.5
BGLAP	AGCGAGGTAGTGAAGAGACGAAAGCCGATGTGGTCAG	55.2
OPN	GCGAGGAGTTGAATGGTGCTTGTGGCTGTGGGTTTC	53.9
ON	GCTGGATGATGAGAACAACACAAGAAGTGGCAGGAAGAG	53.4
CBFA I	GGCAGTTCCCAAGCATTTCGCAGGTAGGTGTGGTGTG	54.5
SOST/sclerostin	CTGGTTAAGAAAGTTGGATAAGAAGGTTACACAGCAAGTTAG	53.8

## Results

### Atomic Force Microscopy (AFM)

**Fig. 1:**
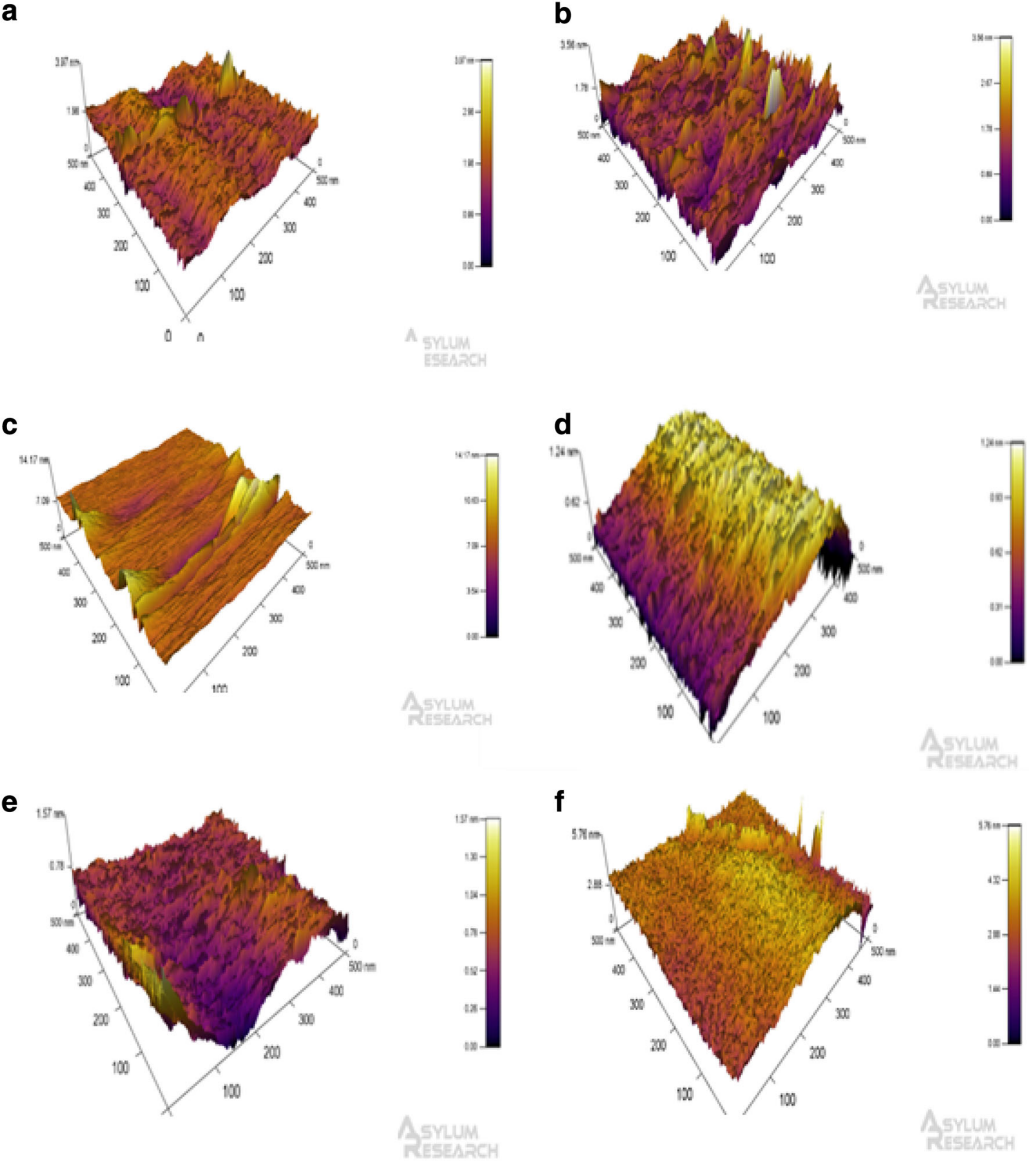
AFM microscopy of different chain length silanes. **a** CL3 modification, **b** CL4, **c** CL6, **d** CL7, **e** CL11 and **f** unmodified glass. Silane modifications were added to glass substrate, and AFM microscopy was performed in tapping mode. AFM microscopy shows the topographical differences seen on the nanoscale

AFM data demonstrated that different topographical profiles were present when different chain lengths were used. The AFM images of CL3 and CL4 (Fig. 1a and b) were similar in height and feature size—both having maximum feature heights of under 10 nm. The CL7 modification (Fig. 1d) showed the formation of clumps of matter which form ridges. This pattern was also seen on the CL6 (Fig. 1c) surface.

### Water Contact Angle Measurements

**Fig. 2:**
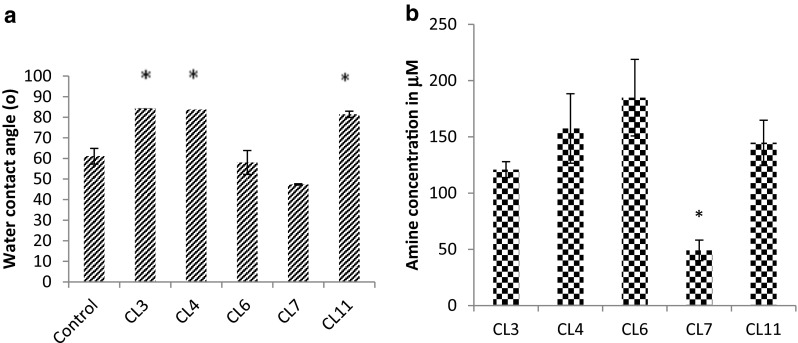
**a** Dynamic water contact angle chart showing dynamic water contact angle of a control glass and glass modified with CL3, CL4, CL6, CL7 and CL11. Stars indicate statistical significance (*p* > 0.05%). **b** Ninhydrin assay for amine concentration chart showing concentration of amine groups on surfaces. This demonstrated that the concentration on CL7 was significantly less than any of the other modifications when analysed using ANOVA (*p* = ≥0.05)

When the variance of mean was examined using ANOVA (*p* ≥ 0.05), CL3, CL4 and CL11 showed a significantly different water contact angle when compared to the control and to CL6 and CL7. CL6 and CL7 did not differ significantly from the control (Fig. 2a).

### Ninhydrin Assay on Films and Glass

The ninhydrin assay (Fig. 2b) demonstrated that amine concentration on CL7 was significantly less than any of the other modifications when analysed using ANOVA (*p* ≥ 0.05).

### Von Kossa Staining Osteoblast-Like Cells on Modified Materials

**Fig. 3:**
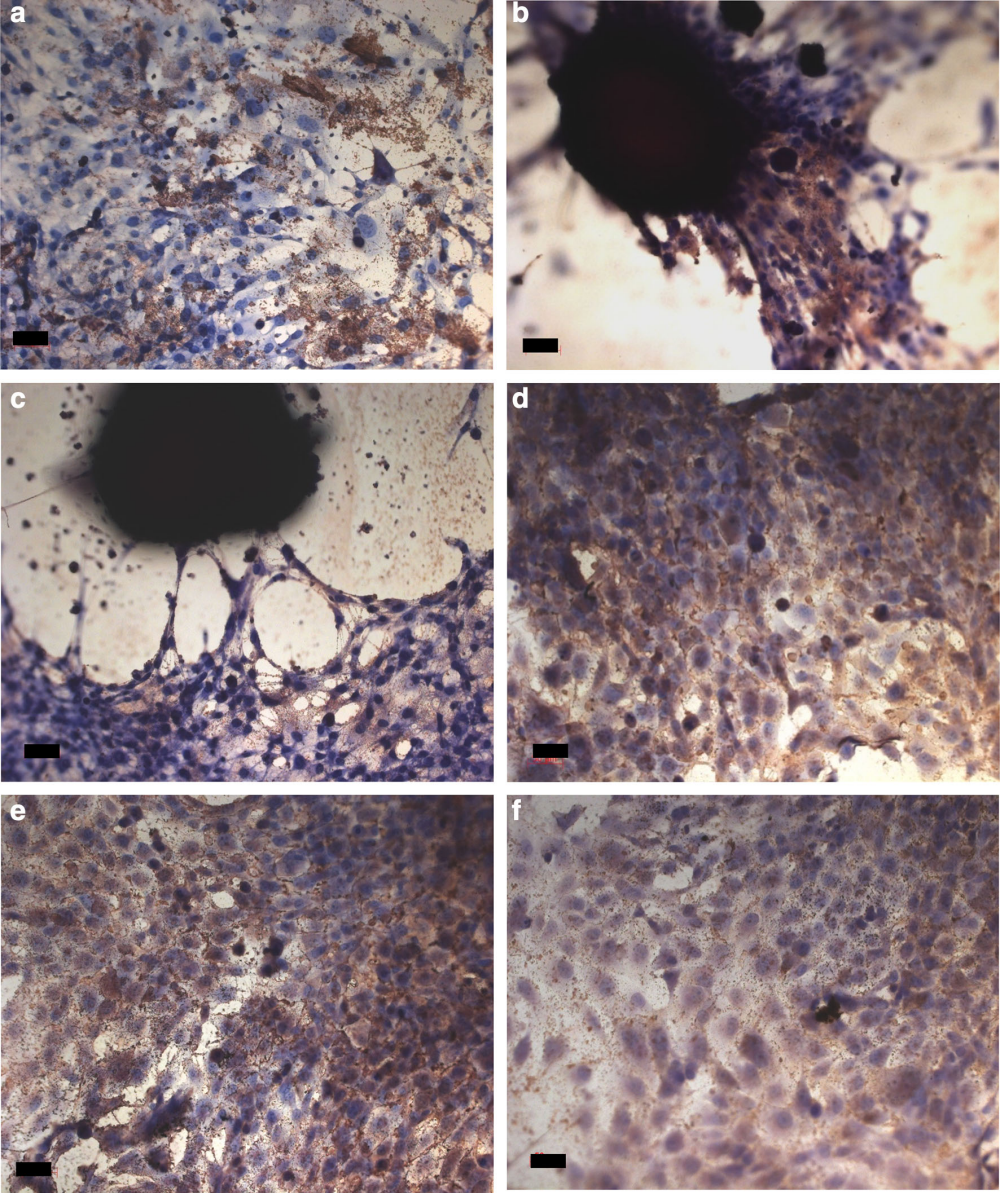
Osteoblast-like cells cultured on silane-modified glass for 7 days. Osteoblast-like cells were cultured on the silane-modified glass (and an untreated control) for 7 days and then stained with von Kossa’s stain for mineralisation **a** untreated glass control, **b** CL3, **c** CL4, **d** CL6, **e** CL7 and **f** CL11

Qualitative evaluation of the cells cultured in contact with the surfaces and stained for the production of a calcified extracellular matrix using von Kossa demonstrated that all surfaces supported osteoblast-like cell adhesion at all the time points tested (Fig. 3). In addition to supporting viable cell adhesion, cells cultured on CL3 and CL4 substrates (Fig. 3b and c) produced calcified nodules at various points on the surfaces by day 7, and this response was not observed on the control or other –NH_2_-modified surfaces. Cells cultured on CL3 and CL4 surfaces maintained their ability to continuously produce calcified nodules throughout the 28-day test period.

### Nodule Count Using Light Microscopy

**Fig. 4:**
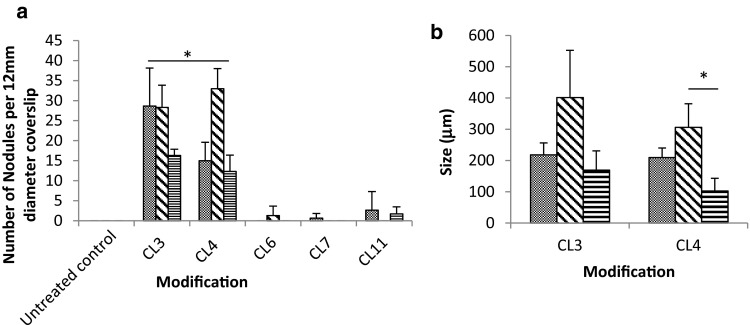
**a** Quantity of nodules formed on the modified surfaces. The nodules were counted using a light microscope. (N = 16) Series 1, 2 and 3 correspond to 7, 14 and 28 days, and results show average and error bars show standard deviation from the mean. **b** Size of nodules on the modified surfaces. Nodules on surfaces treated with CL3 and CL4 were measured after 7, 14 and 28 days; results show average and error bars show standard deviation from the mean

The size and number of cell clusters formed on the CL3 and CL4 substrates were significantly higher than the cell clusters formed on any of the other modifications. The numbers of nodules on each sample were counted by light microscopy (Fig. 4a).

### Scanning Electron Microscopy and Nodule Measurement

**Fig. 5:**
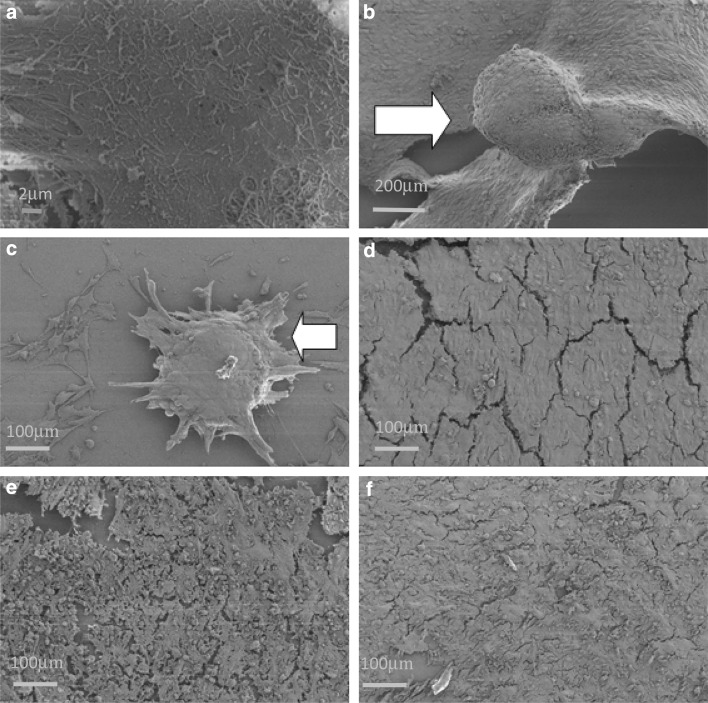
SEM micrographs of osteoblast-like cells cultured on silane-modified glass after 28-day incubation. Osteoblast-like cells were isolated from human trabecular bone and seeded onto the silane-modified surfaces. **a** Untreated glass, **b** CL3, **c** CL4, **d** CL6, **e** CL7 and **f** CL11. White arrows indicate nodules

Cellular interaction with the control and modified substrates was further evaluated using SEM analysis (Fig. 5). In line with previous observations, representative SEM micrographs demonstrated that all test substrates supported cell monolayer attachment and the production of an ECM. Once again, there was evidence of the formation of cell clusters forming on CL3 and CL4 substrates (Figs. 5b and c). SEM micrographs clearly show that the cell clusters are encased in a cohesive ECM, supporting the definition of calcified cell nodules. This phenomenon was not observed on any other test or control substrate at any time point. The size of nodules was measured using the measuring facility on the SEM software (Zeiss SEM user interface) (Fig. 4b), increased significantly by the 14-day period on the CL4 modification and then reduced significantly by 28 days.

### Real-Time Polymerase Chain Reaction

**Fig. 6:**
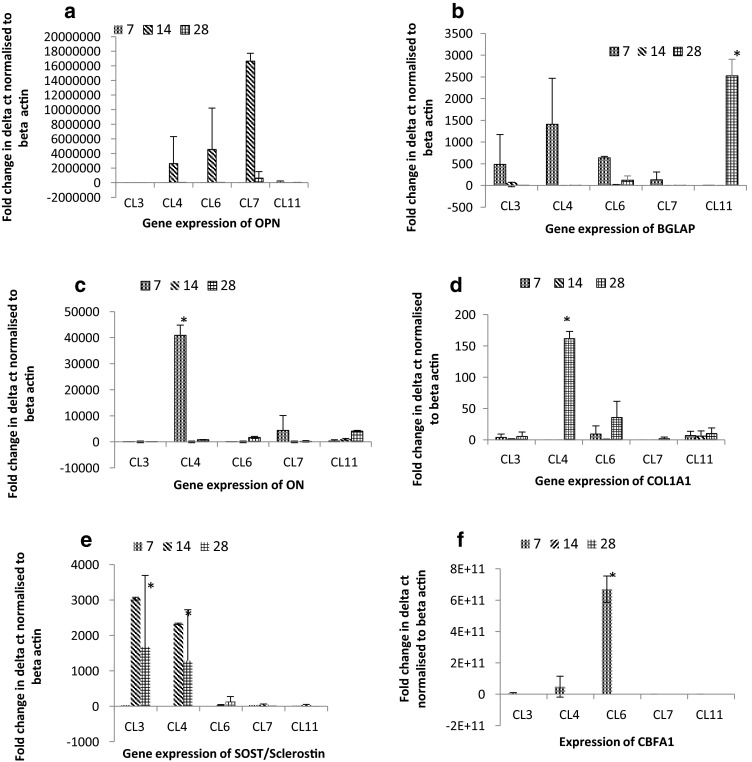
Expression of OPN (**a**), BGLAP (**b**), ON (**c**), COL1A1 (**d**) and SOST/sclerostin (**e**) and CBFA-1 (**f**) in human osteoblast-like cells after 7-, 14- and 28-day incubation with silane-modified glass. Osteoblast-like cells were isolated from human trabecular bone and processed for RT-PCR. Expression of osteopontin was measured and normalised to expression of β-actin and unmodified scaffold. Data shown are average expression and standard deviation from mean. * *p* < 0.10, ** *p* <0.05, *** *p* < 0.01

The data are presented as an up-regulation in activity per cell compared to the glass control. Cells cultured on CL3 surfaces did not show a sustained up-regulation of osteogenic markers, compared to the glass control at all the time points. In contrast, cells cultured on CL4-modified substrates demonstrated an up-regulation of OPN (day 14), BGLAP (day 7), ON (day 7) and COL1A1 (day 28). Cells cultured on both CL3 and CL4 substrates demonstrated a sustained up-regulation of SOST/sclerostin from day 14 (Fig. 6e). This was statistically significant on both CL3 and CL4 surfaces at day 14. Cells cultured on CL6-, CL7- and CL11-modified substrates did not demonstrate a sustained up-regulation of SOST/sclerostin of the same magnitude as CL3 and CL4 substrates. Cells cultured on CL6, CL7 and CL11 substrates again did not demonstrate a sustained up-regulation of osteogenic markers throughout the test period, except the expression of BGLAP on CL11 at day 28, which was significantly enhanced. This finding was in line with the enhanced ECM observed in Fig. 5f. CBFA-1 was expressed on CL6, but not the other modifications, and this indicated that the cells cultured on CL6 were at an earlier stage of osteogenesis than the other modifications.

### Lactate and Glucose Measurement in Culture Media (Results in Supplementary Materials)

Lactate and glucose depletion were indicative of the proliferation [16] of all the cells during the 28-day culture period, demonstrating the cells were healthy in all of the samples/modifications, and that the results seen in the CL3 and CL4 were not different to the other modifications because the other modifications had a detrimental effect on the cells.

## Discussion

Previous work has evaluated the potential of silane-modified substrates, specifically –NH_2_-modified substrates as a tool for the osteoinduction of MSCs. The data from these studies demonstrated that silane modification can be used to induce changes to material surface properties at the submicron scale, combining the optimal parameters of surface chemistry and nanotopography, which in turn can be used to control initial cell adhesion and ultimate cell response [5, 11, 17–20]. To date, the effect of these substrates on differentiated cell types, i.e. osteoblasts, has not been established nor has the optimal combination of material chemistry and nanotopography required to enhance osteoblast activity [21–23]. To optimise the potential of –NH_2_ silane-modified surfaces in both in vitro osteoblast culture, and as coatings for orthopaedic devices, the effect of –NH_2_-enriched surfaces with controlled nanotopography must be understood.

The material properties of the surfaces were altered significantly following silane modification and clearly demonstrated by the change in water contact angle and the concentration of amine groups associated with each of the test substrates. The variables were reproducible on all surfaces and dependent on the chain length used to produce the monolayer. Results demonstrated that the amine concentration on CL7 was significantly less than on any other modification. When taken in conjunction with data derived from the ninhydrin assay, there was a correlation between the significantly lower concentration of amine on the CL7 surface and the significantly reduced WCA. These results suggest that the concentration of surface amine is responsible for the observed changes to water contact angle. The reduction in amine concentration and associated WCA on CL7 could be attributed to amine chain clustering during the silanisation process, where a complete self-assembled monolayer (SAM) did not form. This hypothesis was further strengthened by the presence of clumps of matter which form relatively (from a nanotopography standpoint) large ridges (Fig. 1d) on the AFM micrographs. The presence of clumps of material would result in a reduction in surface stability, which are unlikely to withstand the vigorous washing procedure associated with surface characterisation techniques [24]. These results suggest that the CL7 modification is inappropriate for cell culture, or in vivo implantation, as the stability of the coating cannot be verified.

AFM highlighted the differences in nanotopography (Fig. 1) and demonstrated that there were differences between the various silanes, supporting the hypothesis that CL3 (1a) and CL4 (1b) offer consistent surface roughness.

Taken together, the three characterisation techniques (AFM, ninhydrin and WCA) demonstrated that the silanes used in this study successfully modified the glass substrate. They created different surface topographies when examined on the nanoscale, in addition to a change in surface chemistry. There was an increased concentration of amine groups following modification. Amine groups have been demonstrated in previous studies to show osteogenic capacity [7, 25]. Most previous research has focused on the influence of surface nanotopography on stem cells, and it has been demonstrated that there is an optimum surface topography for osteogenic differentiation [26]. The key conclusion from studies that were successful in creating osteogenic topographies was that a non-ordered surface roughness was optimal.

The von Kossa staining of osteoblasts revealed several significant effects. There was a response on the CL3- and CL4-treated surfaces which lead to the formation of mineralised nodules by 7 days. This response was maintained throughout the culture period of 28 days and was limited to the CL3 and CL4 modifications. A statistically insignificant number of nodules were observed on the other modifications which supported cell expansion throughout the culture period, but with no observed change in phenotype during this time. The number of nodules was counted visually, and the resulting data are displayed in Fig. 4a. The number of nodules was significantly reduced at 28 days on both CL3 and CL4, compared to that at 14 days, analysed by *T* test. There was a significant increase on CL4 between 7 and 14 days, whereas there was a plateau between 7 and 14 days on CL3. Nodule formation is an important process in the life cycle of the osteoblast—it is one of the main defining factors that distinguish between osteoblastic and osteocytic activity. The presence of nodules is a clear indicator of the continued differentiation of these cells.

**Fig. 7:**
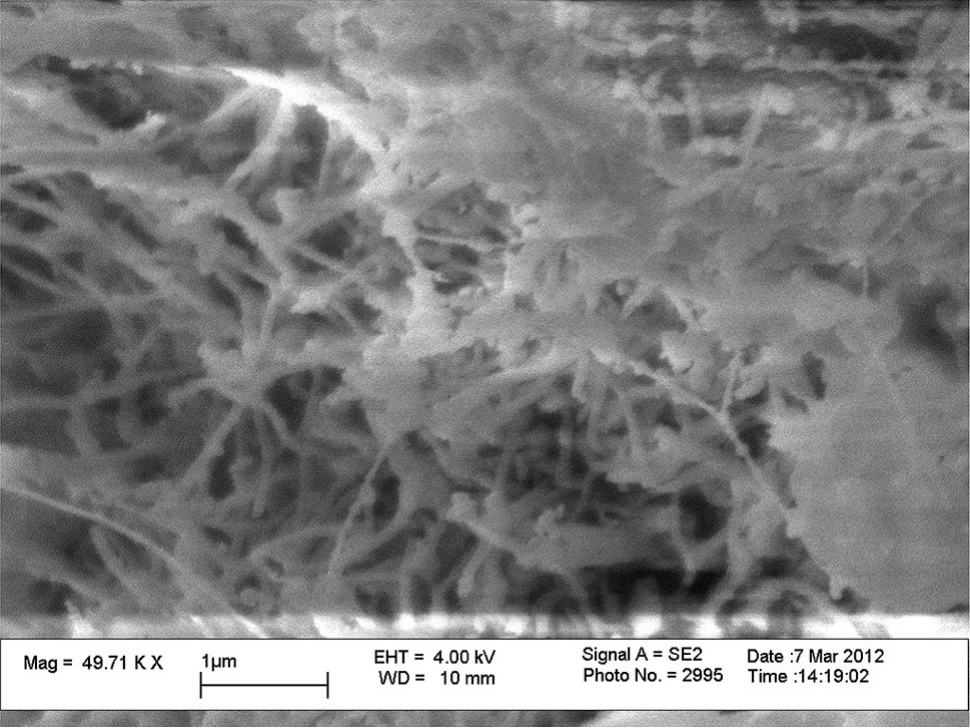
High-magnification image of the nodule formed on CL3 modification. Image shows very tight structure of protein fibres, encasing osteocyte cells which express the sclerostin gene at the mRNA level

The SEM images (Fig. 5) clearly demonstrated nodule formation. The nodules on CL3 (Fig. 5b) and CL4 (Fig. 5c) appeared to be covered in ECM (Fig. 7), which matured with time. This mineralisation was also demonstrated by the positive von Kossa staining from early time points in the culture period. ECM was also produced in abundance on the CL11 surface, but in the absence of nodule formation to any significant degree. SEM analysis of the nodule samples—further higher-magnification images included (Fig. 7)—shows that the cells are producing a heavily mineralised matrix which is only present on the samples which show sclerostin/SOST gene expression at mRNA level.

The primary human osteoblast-like cell model we utilised represents an advanced stage in the osteogenic pathway. Examining how these cells interact with the modified surfaces was conducted to demonstrate how mature terminally differentiated cells react to the surface modifications, providing an insight to the potential longer-term effects of implantation. There was an interesting response observed at the first time point: von Kossa staining (Fig. 3) demonstrated mineralised nodule formation on the CL3 and CL4 surfaces. This reaction was not observed on the other surfaces, where the cells remained in monolayer throughout the 28-day period. Nodule formation on CL3 and CL4 could be explained by the progression of the osteogenic differentiation of the cells. Primary human osteoblasts have been shown to form nodules [22] when in 3D culture on bioactive glass, but only in the presence of exogenous growth factors. The stimulus we observed derived purely from the modified material.

The formation of nodules on CL3 and CL4 surfaces was confirmed by SEM and von Kossa staining. As the cells used in this model were already capable of producing ECM, all of the samples including the untreated glass control stained positive for mineralisation (von Kossa) at 7 days; however, the cells on the untreated control were less densely mineralised by 28 days than the silane-treated samples. The cells on CL6, CL7 and CL11 all retained the ability to produce matrix which became mineralised.

The formation of nodules supports the hypothesis that a population of the cells were starting to gain the characteristics of osteocytes. The embedding of the cells in a highly dense matrix is an important physical step along the pathway towards becoming osteocytes, which have been shown to be important in the regulation of the osteogenic process.

The size of the nodules (Fig. 4b) was shown to increase over a 14-day period and then significantly decrease. This occurrence, when combined with the statistically significant drop in the total number of nodules (Fig. 4a) at 28 days, could be indicative of the nodules reaching a critical size and then detaching from the surface. Interestingly, on CL3 and CL4 surfaces the expression of sclerostin was notable at 14 and 28 days. Sclerostin is a Wnt antagonist and is specifically a marker of osteocytic activity [13]. Along with the reduction of normal osteoblastic markers after 7 days, this is indicative of the further differentiation of the cells along the osteogenic pathway. All the results are consistent with the hypothesis that the materials can influence osteoblast-like cell fate and cause them to express osteocyte-specific markers. Sclerostin is only expressed on the materials (CL3 and CL4) which induce nodule formation, indicating that it is specific to cells embedded in matrix, which is consistent with current opinion that sclerostin is a marker specific to embedded osteocytes. The differences observed between the chain lengths and their osteogenic effects can be attributed to surface topography that is induced at the nanoscale and the mimicry of the correct ECM by the chemical modification. In the instance of the CL3 and CL4 nodule formation, it is possible that the amine-rich surfaces and the topography instigated the formation of nodules and the expression of osteocytic markers.

This work leads to the conclusion that it is possible to use inexpensive surface chemistry and topography modifications to mimic the role of ECM in the osteogenic differentiation pathway. This ultimately could lead to a synthetic, economically viable off-the-shelf material suitable for bone regeneration applications in currently difficult-to-treat medical conditions.

## Electronic supplementary material

Below is the link to the electronic supplementary material.

Supplementary material 1 (DOCX 1046 kb)

Supplementary material 2 (DOCX 1188 kb)

Supplementary material 3 (DOCX 19 kb)

Supplementary material 4 (DOCX 19 kb)
