# Supplementary material for: Defining the Properties of an Array of –NH2-Modified Substrates for the Induction of a Mature Osteoblast/Osteocyte Phenotype from a Primary Human Osteoblast Population Using Controlled Nanotopography and Surface Chemistry
Source: Calcif Tissue Int. 2016 Oct 28;100(1):95–106. doi: 10.1007/s00223-016-0202-y (PMC5214888; doi:10.1007/s00223-016-0202-y)
Supplement: Supplementary file 2 — Supplementary material 2 (DOCX 1188 kb) [file 223_2016_202_MOESM2_ESM.docx]

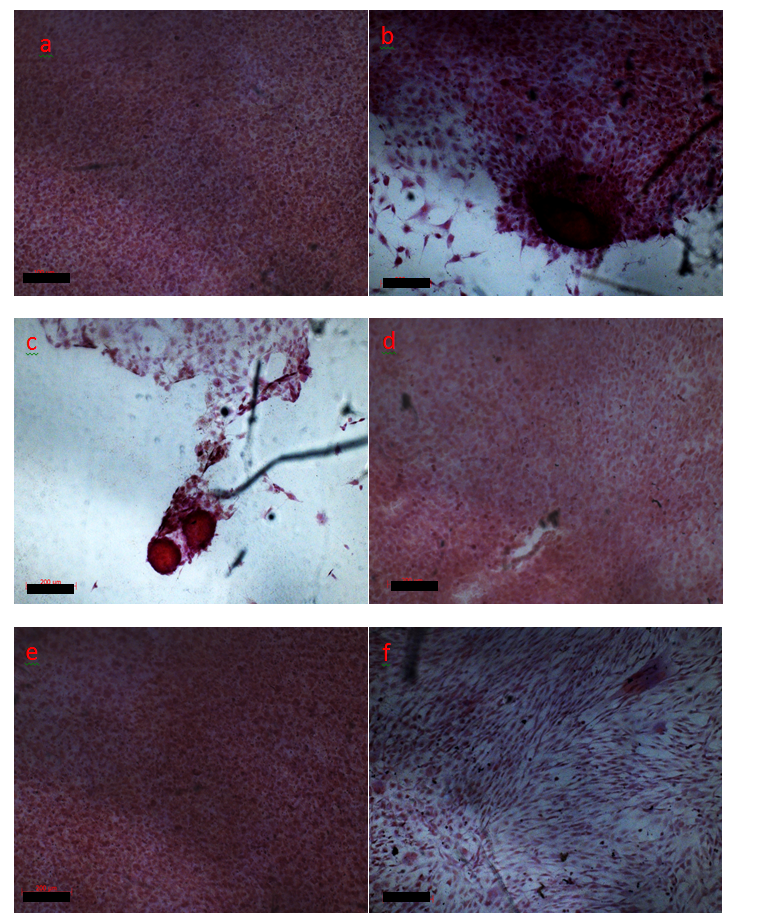


**Supplementary material 3: Osteoblast-like cells cultured on silane modified glass for 28 days.** Osteoblast like cells were cultured on the silane modified glass (and an untreated control) for 28 days, then stained with Von Kossa’s stain for mineralisation (a) untreated glass control, (b) CL3, (c) CL4, (d) CL6, (e) CL7 and (f) CL11. All scale bars equate to 200μm
