# Supplementary material for: Defining the Properties of an Array of –NH2-Modified Substrates for the Induction of a Mature Osteoblast/Osteocyte Phenotype from a Primary Human Osteoblast Population Using Controlled Nanotopography and Surface Chemistry
Source: Calcif Tissue Int. 2016 Oct 28;100(1):95–106. doi: 10.1007/s00223-016-0202-y (PMC5214888; doi:10.1007/s00223-016-0202-y)
Supplement: Supplementary file 4 — Supplementary material 4 (DOCX 19 kb) [file 223_2016_202_MOESM4_ESM.docx]

Supplementary material 4: Measurement of glucose in culture media taken from wells of primary osteoblast cultures with modified materials at 7, 14 and 28 days
